# Gene-Specific Outcomes After Central Nervous System Metastases in Germline BRCA1- and BRCA2-Associated Breast Cancer

**DOI:** 10.3390/cancers18081240

**Published:** 2026-04-14

**Authors:** Alice Decaminada, Raute Sunder-Plassmann, Weang-Kee Ho, Daniela Muhr, Angelika M. Starzer, Anna Sophie Berghoff, Rupert Bartsch, Christian F. Singer, kConFab Investigators, Yen Y. Tan

**Affiliations:** 1 Department of Obstetrics and Gynecology, Comprehensive Cancer Center, Medical University of Vienna, 1090 Vienna, Austriachristian.singer@meduniwien.ac.at (C.F.S.); 2 Department of Laboratory Medicine, Medical University of Vienna, 1090 Vienna, Austria; raute.sunder-plassmann@meduniwien.ac.at; 3 Faculty of Medical and Life Sciences, Sunway University, Petaling Jaya 47500, Selangor, Malaysia; 4 Cancer Research Malaysia, Subang Jaya 47500, Selangor, Malaysia; 5 Department of Medicine I, Division of Oncology, Medical University of Vienna, 1090 Vienna, Austria; 6 The Peter MacCallum Cancer Centre, Melbourne, VIC 3010, Australia; 7 Sir Peter MacCallum Department of Oncology, University of Melbourne, Parkville, VIC 3010, Australia

**Keywords:** breast cancer, CNS metastasis, brain metastasis, overall survival, BRCA1, BRCA2

## Abstract

**Background:** Many studies evaluating central nervous system (CNS) metastases in breast cancer (BC) combine germline *BRCA1* and *BRCA2* pathogenic variant carriers, limiting gene-specific interpretation. We evaluated gene-specific overall survival (OS) after CNS metastasis and time to CNS involvement. **Methods:** We retrospectively identified BC patients with confirmed CNS metastases (1995–2022) from the Medical University of Vienna and the kConFab consortium. Germline status was classified as g*BRCA1* PV, g*BRCA2* PV, or non-carrier. Primary endpoint was OS from CNS metastasis diagnosis; secondary endpoint was CNS metastasis-free interval from primary BC diagnosis. Kaplan–Meier/log-rank tests were used for group comparisons. Multivariable Cox models assessed OS in complete cases, stratified by molecular subtype and adjusted for prognostic factors. Sensitivity analyses included subtype-adjusted and time-period models. **Results**: Among 115 patients (g*BRCA1* n = 32, g*BRCA2* n = 18, and non-carriers n = 65), median OS differed by germline status (*p* = 0.019): 20.0 months (95% CI 6.7–60.0) for g*BRCA2* versus 7.1 months (95% CI 3.7–10.0) for g*BRCA1* and 7.6 months (95% CI 3.4–12.0) for non-carriers. In subtype-stratified analyses, g*BRCA1* showed similar mortality to non-carriers (HR 0.90, 95% CI 0.49–1.64, *p* = 0.730), while g*BRCA2* showed a lower but non-significant hazard (HR 0.48, 95% CI 0.18–1.25, *p* = 0.131). Median CNS metastasis-free interval was longer for g*BRCA2* (8.4 years) versus g*BRCA1* (3.0 years) and non-carriers (3.1 years; *p* = 0.020). Sensitivity analyses were consistent. **Conclusions**: g*BRCA2* carriers demonstrated longer unadjusted OS after CNS metastasis and a longer CNS metastasis-free interval compared with g*BRCA1* carriers and non-carriers. However, these associations were attenuated and not statistically significant after adjustment, and should therefore be interpreted as hypothesis-generating. These findings supports further investigation of gene-specific CNS disease trajectories in larger cohorts.

## Simple Summary

Breast cancer is one of the leading causes of central nervous system (CNS) metastases, which are associated with reduced overall survival. In patients with inherited *BRCA1* or *BRCA2* mutations, breast cancers differ in their biology and clinical behavior, yet these groups are often combined in studies of CNS disease. In this multicenter cohort from Austria and Australia, we evaluated outcomes separately for *BRCA1* and *BRCA2* mutation carriers and compared them with non-carriers. We found that patients with BRCA2 mutations developed CNS metastases later and showed longer survival after CNS diagnosis in unadjusted analyses. However, these differences were attenuated after accounting for tumor subtype and other clinical factors. These findings suggest that *BRCA1*- and *BRCA2*-associated breast cancers may follow distinct disease trajectories in the CNS setting, but should be interpreted with caution. Our results highlight the importance of gene-specific analyses and support further investigation in larger, prospective cohorts to better understand how inherited genetic background may influence prognosis and treatment in this setting.

## 1. Background

Central nervous system (CNS) metastases, including parenchymal brain metastases and leptomeningeal disease, occur in about 20–30% of patients with metastatic breast cancer (BC) and remain a major cause of morbidity and mortality, with median survival typically ranging from 6 to 36 months depending on tumor subtype and treatment [1,2,3,4,5]. The risk of CNS involvement is strongly influenced by tumor biology and is highest in triple-negative and HER2-positive disease, with important implications for systemic treatment selection. Current management is multidisciplinary and may include stereotactic radiosurgery (SRS) and, in selected cases, surgical resection with systemic treatment tailored to subtype [6,7,8,9]. Whole-brain radiotherapy (WBRT) is now used more selectively because of its adverse effects on neurocognitive function and quality of life. In parallel, contemporary guidelines increasingly support an initial systemic therapy approach in selected patients when subtype-specific agents with demonstrated intracranial activity are available [2,4,5,8,10].

For HER2-positive BC, tucatinib in combination with trastuzumab and capecitabine has been shown to improve intracranial progression-free survival (PFS) and overall survival (OS) [11], while trastuzumab-deruxtecan (T-DXd) has also demonstrated meaningful intracranial activity [12,13,14]. In germline *BRCA1/2 (*g*BRCA1/2)*-associated BC, PARP inhibitors have shown superior efficacy to chemotherapy in metastatic disease [15,16,17,18], with evidence of activity also in patients with stable CNS metastasis [19,20]. However, the impact of g*BRCA1/2* status on outcomes after CNS metastasis remains unclear. Interpretation is complicated by the close relationship between *BRCA* gene status, tumor subtype and baseline CNS risk [21,22,23,24]. In addition, homologous recombination deficiency may influence metastatic patterns, including CNS involvement [25,26].

Although prior studies suggest a higher risk of CNS involvement in g*BRCA1*-associated BC [27,28,29,30] and possible subtype-independent CNS involvement in g*BRCA2*-associated disease [29], CNS-specific outcome data remain limited. Existing studies are constrained by small sample sizes, heterogeneous endpoints, and importantly, the frequent aggregation of *BRCA1* and *BRCA2* carriers, which may obscure clinically meaningful gene-specific differences [29,31].

To address this gap, we analyzed a binational multicenter cohort from Austria and Australia comprising BC patients with CNS metastases to compare OS after CNS metastasis and the CNS metastasis-free interval across g*BRCA1*, g*BRCA2* and non-carriers, while accounting for molecular subtype and key clinical prognostic factors. We hypothesized that g*BRCA2* PV carriers would show distinct CNS disease trajectories compared with g*BRCA1* carriers.

## 2. Methods

### 2.1. Study Design and Population

We conducted a retrospective cohort study of BC patients from the MUV and the national Australasian kConFab consortium with confirmed CNS metastasis (brain parenchymal and/or leptomeningeal disease) diagnosed between 1995 and 2022. CNS involvement was confirmed by computer tomographic and/or magnetic resonance imaging, lumbar puncture or autopsy report. Patients were included in the study if they were 18 years and above, had histologically confirmed BC, known g*BRCA1/2* status (g*BRCA1*/2 PV class 4 or 5 vs. non-carrier class 1 or 2), and provided research consent. Patients were excluded if they had unconfirmed CNS disease, missing genetic status, g*BRCA1/2* class 3 variants (variants of uncertain significance), or non-*BRCA1/2* PVs. Due to low number of patients with HER2-positive and unknown molecular subtypes, these groups were retained for descriptive analyses but were excluded from Cox models.

### 2.2. Variables and Data Collection

MUV clinical data were extracted from medical or autopsy records. Germline *BRCA1/2* status was derived from clinical genetic testing and retrieved from the Austrian ATHENA hereditary cancer registry, and outcomes data through linkage with Statistics Austria. kConFab data were collected through treating hospitals, clinical pathology centers and cancer registries with additional information obtained from structured follow-up questionnaires administered every three years. In the present analyses, “non-carriers” refers to non-carriers of g*BRCA1/2* pathogenic variants. The extent of additional multigene panel testing differed by cohort and calendar period. In the MUV cohort, panel testing beyond *BRCA1/2* was introduced from 2015 onwards, whereas in kConFab, a subset of *BRCA1/2*-negative individuals underwent at least a 10-gene panel test. As such, undetected pathogenic variants in other homologous recombination repair genes (e.g., PALB2, ATM) cannot be fully excluded in earlier testing eras.

Variables collected included sex assigned at birth, menopausal status at BC diagnosis, tumor histology/molecular subtype, local CNS treatments, presence of leptomeningeal disease or other metastases detected by imaging, CNS lesion count, and CNS-directed treatment types.

### 2.3. Statistical Analysis

Baseline characteristics were summarized by g*BRCA* status using medians and interquartile ranges (IQRs) for continuous variables and counts with percentages for categorical variables. Group comparisons were performed using a Kruskal–Wallis test for continuous variables and Chi-square or Fisher’s exact tests for categorical variables.

OS was defined as the time from CNS metastasis diagnosis to death from any cause and patients alive at last follow-up were censored. The primary endpoint was OS from CNS metastasis diagnosis. Secondary endpoint was CNS metastasis-free interval, defined as the time from primary BC diagnosis to CNS metastasis. We estimated OS and CNS metastasis-free interval using Kaplan–Meier methods and compared germline groups using log-rank tests, with Bonferroni-adjusted pairwise comparisons. We did not fit multivariable models for this endpoint because all included patients developed CNS metastasis by design and it does not allow valid inference regarding predictors of incident CNS involvement in the broader metastatic population. In addition, several factors such as imaging intensity/surveillance and systemic therapy exposure are not captured with sufficient completeness to support a robust multivariable risk model.

Multivariable associations with OS were evaluated using Cox proportional hazards regression. Prespecified covariates included age at CNS metastasis diagnosis (continuous), g*BRCA* status (g*BRCA1* PV, g*BRCA2* PV, non-carrier), presence of leptomeningeal disease (yes/no), number of CNS metastases (1/≥2), presence of extracranial metastases (yes/no), and receipt of local CNS-directed therapy (WBRT-only, SRS-only, WBRT + SRS, any surgery ± WBRT/SRS, and no local therapy). Covariates were selected based on clinical relevance and established prognostic factors for BC brain metastases [1]. We could not implement the diagnosis-specific graded prognostic assessment (DS-GPA) score in the models because performance status (ECOG or Karnofsky) was not available consistently for both cohorts. We therefore adjusted for key prognostic components aligned with those used in DS-GPA that were captured in our dataset, including germline status. Molecular subtype was additionally addressed through stratified analyses and sensitivity-adjusted models to evaluate the robustness of estimates to residual confounding by subtype. Cox models were restricted to cases with known subtype and limited to luminal and triple-negative cases only. HER2-positive cases were excluded because of low numbers (n < 5) to ensure model stability and to avoid unreliable estimates. All analyses were conducted as complete-cases analyses without imputation. Proportional hazards assumptions were evaluated using Schoenfeld residuals. To evaluate whether results were sensitive to temporal changes in CNS imaging and clinical management, we repeated the models using a time-period indicator (diagnoses pre- and post-2010). Year 2010 was selected to approximate a shift toward routine MRI availability and modern stereotactic approaches. Potential cohort effects were explored by comparing OS between cohorts using Kaplan–Meier curves and log-rank tests. Cohort-stratified Cox models were not fitted due to small sample size, but cohort-specific univariable models were estimated to assess whether the direction of *gBRCA* associations was consistent within each cohort. All analyses were performed using R (version 4.4.1) and two-sided *p*-values < 0.05 were considered statistically significant.

## 3. Results

### 3.1. Descriptive Analysis

**Figure 1:**
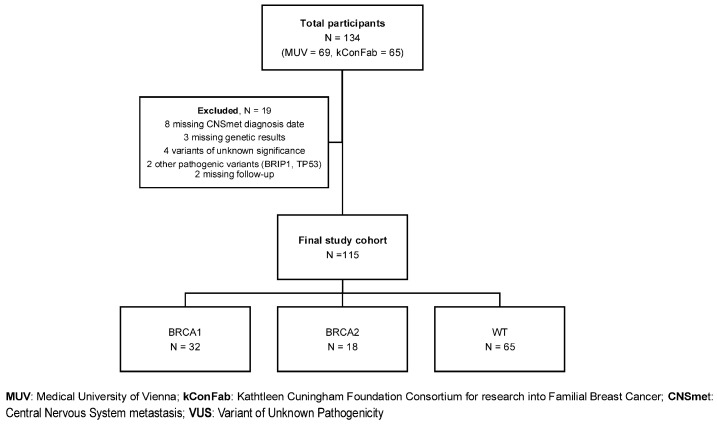
Study cohort inclusion.

Figure 1 shows patient disposition. Baseline characteristics by g*BRCA1* status are presented in Table 1. Of 115 patients, 105 were female, 3 were male, and 7 unspecified. Median age at CNS metastasis diagnosis was 48.4 years (IQR 40.3–56.3) and differed significantly across g*BRCA* subgroups (*p* < 0.003), with g*BRCA1* PV carriers presenting at a younger age than both g*BRCA2* PV carriers and non-carriers (41.1 years vs. 48.9 years vs. 51.6 years, respectively). Bonferroni-adjusted pairwise comparisons showed differences between g*BRCA1* and g*BRCA2* PV (*p* = 0.017) and between g*BRCA1* PV and non-carriers (*p* = 0.011), but no difference was observed between g*BRCA2* PV and non-carriers (*p* = 0.999). The younger age at CNS metastasis among g*BRCA1* carriers was also observed when restricting descriptively to triple-negative cases. Breast cancer molecular subtype showed borderline differences by g*BRCA* status (*p* = 0.053), with g*BRCA1* PV carriers enriched for triple-negative disease (65.6%) and g*BRCA2* PV carriers enriched for luminal disease (44.4%). Subtype was unknown more often among gBRCA2 PV carriers (38.9%). When restricted to cases with known subtype, baseline distributions also differed between MUV and kConFab cohorts (Supplementary Table S1), so cohort effects on OS were assessed prior to pooling.

### 3.2. Survival Analysis

During the observation period, 104/115 patients died and eleven were censored alive at last follow-up, giving an overall event rate of 90.4%. Among censored patients, median follow-up was 16.5 months (IQR 4.3–38.5).

**Figure 2:**
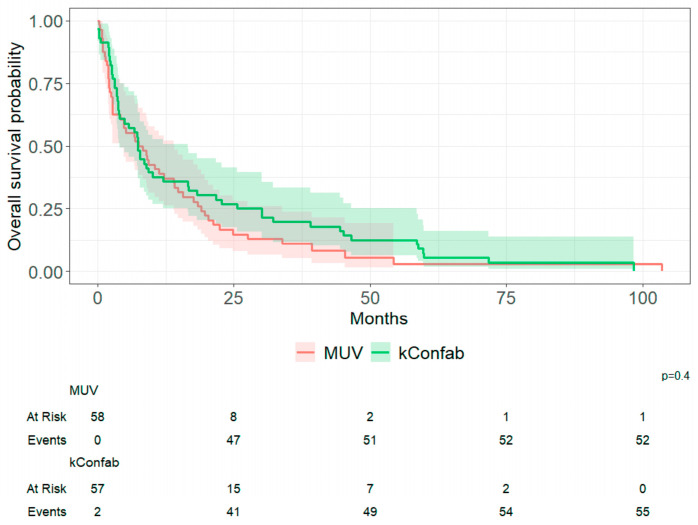
Overall survival after CNS metastasis by cohort.

**Figure 3:**
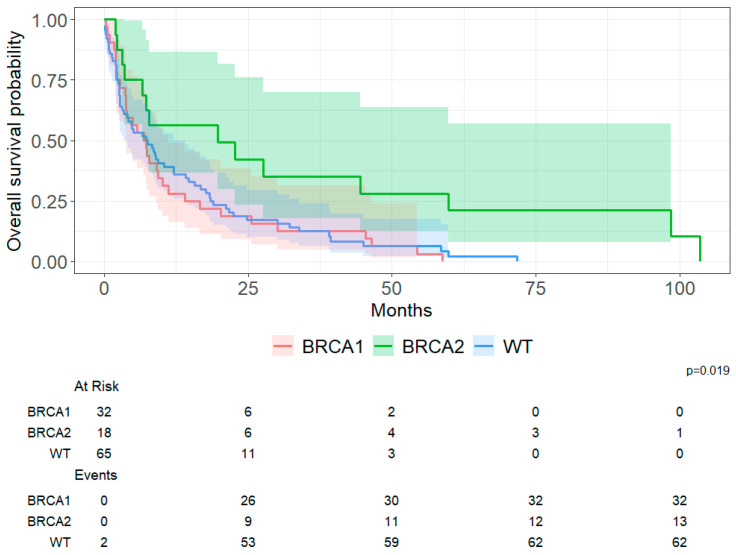
Overall survival after CNS metastasis categorized by g*BRCA* status.

OS did not differ between the MUV and kConFab cohorts on Kaplan–Meier analysis (Figure 2). Cohort-specific Kaplan–Meier curves across germline groups are shown in Supplementary Figures S1 and S2. Cohort-specific univariable Cox models are provided in Supplementary Table S2. Cohort-specific estimates for g*BRCA2* carriers versus non-carriers were consistent in direction, although confidence intervals were wide due to small sample size (MUV HR = 0.26, 95% CI 0.06–1.09, *p* = 0.065; kConFab HR = 0.62, 95% CI 0.29–1.33, *p* = 0.220). As such, subsequent analyses were performed using the combined dataset. Figure 3 shows that survival differed across g*BRCA* subgroups. Germline *BRCA2* PV carriers had the longest post-CNS metastasis survival (median 20 months; 95% CI 6.7–60.0) compared with g*BRCA1* PV carriers (7.1 months; 95% CI 3.7–10.0, *p* = 0.051) and non-carriers (7.6 months; 95% CI 3.4–12.0, *p* = 0.029). The overall three-group comparison was significant (log-rank *p* < 0.019). In Bonferroni-adjusted pairwise comparisons, survival differed between g*BRCA2PV* carriers and non-carriers (*p* = 0.029) while the comparison between g*BRCA2* and g*BRCA1* PV carriers was borderline (*p* = 0.051).

To contextualize these findings, we also derived absolute survival estimates from the Kaplan–Meier curves. At 12 months after CNS metastasis diagnosis, approximately 56% of g*BRCA2* PV carriers remained alive compared to 39% of non-carriers and 28% of g*BRCA1* PV carriers. This represents a 17% absolute difference in 1-year survival between g*BRCA2* PV carriers and non-carriers.

### 3.3. Multivariate Analysis

In a multivariable model stratified by subtype (n = 77, restricted to luminal and triple-negative subtype, Table 2), g*BRCA2* PV carriers had a lower hazard of death compared with non-carriers (HR = 0.48, 95% CI 0.18–1.25, *p* = 0.131), although this finding did not reach statistical significance. g*BRCA1* PV carriers had hazards like non-carriers (HR 0.90, 95% CI 0.49–1.64, *p* = 0.730). Subtype effects were not estimated directly in this model due to stratification. Compared to WBRT alone, patients who received SRS only (HR = 0.55, 95% CI 0.23–1.30, *p* = 0.175), WBRT + SRS (HR = 0.57, 95% CI 0.26–1.28, *p* = 0.173), and any surgery ±WBRT/SRS (HR = 0.58, 95% CI 0.26–1.25, *p* = 0.164) had lower hazards, whereas no local therapy had a higher hazard (HR = 2.28, 95% CI 0.84–6.16, *p* = 0.105). Schoenfeld residuals test did not suggest violations of the proportional hazards assumption for the final subtype-stratified complete-case model.

In the model including molecular subtype as an adjustment covariate (Supplementary Table S3), TNBC was associated with worse OS versus luminal disease (HR = 1.83, 95% CI 1.00–3.36, *p* = 0.050) while the g*BRCA2* estimate continued to favor lower mortality versus non-carriers, although it was not statistically significant (HR = 0.46, 95% CI 0.18–1.17, *p* = 0.104). In time-period sensitivity analyses, the g*BRCA2* association was similar in direction (g*BRCA2* PV vs. non-carrier, HR = 0.55–0.57; Supplementary Tables S4 and S5).

### 3.4. CNS Metastasis-Free Interval

**Figure 4:**
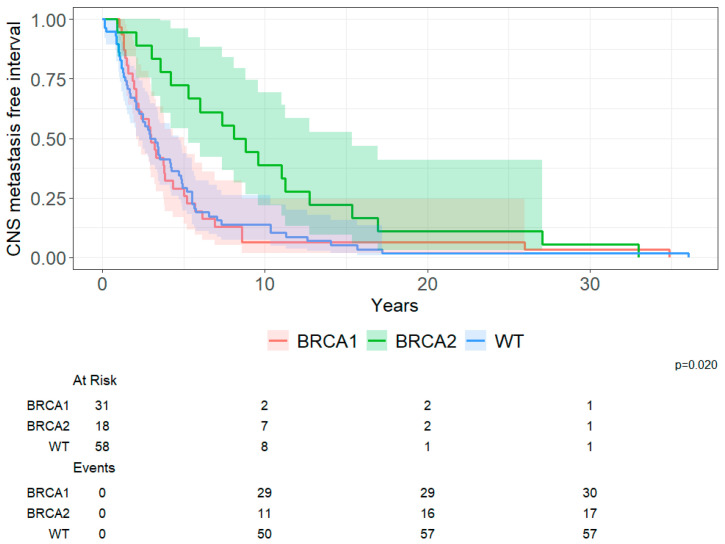
CNS metastasis-free interval by g*BRCA* status.

A total of 107 patients were included in this analysis. Eight were excluded due to missing date of primary BC diagnosis. CNS metastasis-free interval differed by germline group (log-rank, *p* = 0.020; Figure 4). Median CNS metastasis-free interval was 3.0 years for g*BRCA1* PV carriers (95% CI 2.1–5.0), 8.4 years for g*BRCA2* PV carriers (95% CI 5.3–15.0) and 3.1 years for non-carriers (95% CI 2.3–4.7). In the pairwise comparisons, g*BRCA2* carriers had a significantly longer CNS metastasis-free interval than both g*BRCA1* PV carriers (*p* = 0.006) and non-carriers (*p* = 0.006), while no significant difference was observed between g*BRCA1* PV carriers and non-carriers.

## 4. Discussion

In this multicenter retrospective cohort of BC patients with CNS metastases, outcomes differed by germline *BRCA* status, with g*BRCA2* PV carriers showing longer OS post-CNS metastasis than g*BRCA1* PV carriers and non-carriers in unadjusted analysis. This is clinically relevant because g*BRCA1* and g*BRCA2*-associated BC differ in clinicopathologic features and systemic disease course [21,22,23,32], yet many prior studies have pooled g*BRCA1* and g*BRCA2* carriers, which can obscure meaningful gene-specific patterns. Our CNS-specific results are timely and provide context in an era when systemic therapies with intracranial activity, such as PARP inhibitors and antibody–drug conjugates, are increasingly used in metastatic BC with CNS involvement (for, e.g., OlympiAD [15], EMBRACA [17] and DESTINY-Breast12 [12]), while CNS-specific evidence remains comparatively limited [5]. In parallel, molecular profiling studies report higher homologous recombination deficiency (HRD) signals in BC brain metastases than in matched primary tumors, raising the possibility that PARP inhibitor benefit may extend beyond germline *BRCA*-associated disease and supporting prospective evaluation of HRD-guided systemic approaches in CNS metastases [25,33,34].

The apparent g*BRCA2* survival advantage observed in the unadjusted analyses was attenuated and no longer statistically significant in multivariable models, both when molecular subtype was handled by stratification or when it was included as an adjustment covariate. This may reflect, in part, the unequal distribution of tumor subtype across g*BRCA* groups, a major determinant of outcomes after CNS metastasis, with g*BRCA1* cases enriched for TNBC and g*BRCA2* for hormone-receptor positive or luminal disease. The small number of g*BRCA2* cases also limited precision for adjusted estimates. These findings should therefore be interpreted as associations rather than evidence of an independent prognostic effect of g*BRCA* status after CNS metastasis.

A key consideration in interpreting these findings is the strong correlation between g*BRCA* status and breast cancer molecular subtype. In our cohort, g*BRCA1* carriers were predominantly triple-negative, whereas g*BRCA2* carriers were more frequently associated with luminal subtypes. Given the well-established differences in natural history, treatment responsiveness, and survival outcomes between these subtypes, it is likely that part of the observed survival advantage in g*BRCA2* carriers reflects subtype-related effects rather than germline status per se. Although we addressed this through stratified and adjusted analyses, residual confounding cannot be excluded, particularly given the limited sample size.

Our gene-specific findings are consistent with a prior report suggesting g*BRCA2* PV carriers have longer survival after brain metastasis than g*BRCA1* patients [28]. Berliner et al. [31] reported substantially shorter survival in pooled g*BRCA1/2* carriers compared with non-carriers after CNS diagnosis, but the sample size was small (n = 75) and the lack of gene-specific stratification limits interpretation. When we applied the same pooled-carrier approach to our dataset (i.e., g*BRCA1* + g*BRCA2* PV versus non-carriers), survival was similar between carriers and non-carriers (log-rank *p* = 0.295, HR 0.81, 95% CI 0.55–1.20), illustrating how pooling can obscure gene-specific outcomes and contribute to discrepant findings across studies. Song et al. [29] studied patients at first locoregional recurrence and distant metastasis, and found that CNS involvement was frequent among g*BRCA* PV carriers, particularly for g*BRCA2* PV carriers. However, their survival endpoint was BC-specific survival and measured from first recurrence or metastasis instead of CNS diagnosis, which limited direct compatibility with our study. Moreno et al. [27] restricted to patients diagnosed with TNBC, and likewise, reported higher risk of metastasis among g*BRCA2* PV carriers. They also reported a trend toward lower mortality among g*BRCA* carriers compared to non-carriers (g*BRCA1* PV HR 0.52, 95% CI 0.27–1.00, *p* = 0.053; g*BRCA2* PV HR 0.80, 95% CI 0.31–2.09, *p* = 0.659). Their cohort also had more bone metastases in g*BRCA2* PV carriers and lung metastases in g*BRCA1* PV carriers, patterns which we similarly observed in our cohort but did not reach statistical significance. These differences in extracranial metastatic patterns may be clinically relevant, as visceral metastases such as lung involvement are generally associated with poorer prognosis compared with bone-dominant disease. As such, variation in metastatic distribution across germline groups may have contributed to the observed survival differences. However, given the limited sample size and the absence of time-dependent modeling of metastatic burden and progression, we are unable determine whether these patterns independently account for differences in OS across groups. These findings are therefore interpreted as descriptive and hypothesis-generating, and warrant further investigation in larger cohorts with longitudinal modeling of metastatic trajectories.

We assessed the CNS metastasis-free interval as a secondary outcome, and we found that g*BRCA2* PV carriers had a significantly longer time from primary BC diagnosis to CNS metastasis compared to g*BRCA1* and non-carriers. Berliner et al. [31] reported a slightly longer interval among pooled carriers compared to non-carriers, but their pooled analysis did not allow for gene-specific interpretation. In our cohort, the gene-stratified pattern is consistent with known differences in tumor phenotype and overall disease progression patterns between g*BRCA2*- and g*BRCA1*-associated BC, but it should be interpreted as a timing comparison among patients who developed CNS metastasis.

Beyond survival and metastasis-free interval, we found that g*BRCA1* PV carriers were younger at CNS metastasis diagnosis than g*BRCA2* PV and non-carriers, indicating a difference in age distribution at CNS presentation across germline groups in our cohort. This younger age at presentation was also observed when restricting to triple-negative cases. This finding is consistent with the pooled *BRCA* series from Berliner et al. [31], but in contrast with Song et al. [29] and Moreno et al. [27], who reported age at first distant metastasis or breast metastasis instead of age at CNS metastasis, thereby limiting direct comparison with our findings.

We also observed differences in OS after CNS metastasis across therapy categories. These observations should be interpreted with caution given small subgroup sizes and likely influenced by confounding by indication. Eligibility for surgery or other local therapy depends on factors such as disease extent and importantly performance status [7], which was not captured consistently across both cohorts. Although we adjusted for several prognostic markers, we could not apply a formal disease-specific GPA classification. We therefore interpret therapy-category estimates as descriptive rather than causal. Our results highlight the importance of timely CNS diagnosis and multidisciplinary assessment to preserve eligibility for effective local treatment options.

Several limitations should be considered when interpreting our findings. First, the sample size was small, particularly for g*BRCA2* carriers. Pooling MUV and the kConFab cohort increased sample size but introduced heterogeneity. Despite this, the g*BRCA2* association with post-CNS survival was directionally consistent when cohorts were examined separately. Second, because the cohort was restricted to patients with confirmed CNS metastases only, we could not compare baseline characteristics or outcomes to a metastatic cohort without CNS involvement. Third, genetic testing depth differed by cohort and over time. While some g*BRCA1/2*-negative individuals, particularly those within kConFab, underwent multigene panel testing, earlier cases, especially in the MUV cohort, were tested for g*BRCA1/2* only. As such, a proportion of patients classified as non-carriers may harbor PVs in other homologous recombination repair genes (e.g., PALB2, ATM). This potential misclassification would be expected to dilute true differences between groups and bias estimates toward the null, suggesting that the observed gene-specific patterns may be conservative. Fourth, the study covers a long time span during which access to neuroimaging, CNS-directed treatments and systemic therapies changed substantially. When we repeated the analyses using a time-period comparing diagnoses pre- and post-2010, the estimates were similar in direction, but with wide confidence intervals (due to a few patients diagnosed before 2010), and with hazard >1. This likely reflects ascertainment differences rather than treatment effect where MRI detects more aggressive CNS disease. We therefore view the time-period as a rough proxy for how the cohort and diagnostic practices changed over time, and not as a causal effect. Differences in imaging practices between centers and across time periods may also have introduced detection bias. MRI was more frequently used in the MUV cohort, whereas CT-based diagnosis was more common in earlier periods and in parts of the kConFab cohort. Given the higher sensitivity of MRI for detecting CNS metastases, this may have led to earlier detection and an apparent prolongation of survival following CNS diagnosis. While the time-period sensitivity analyses partially address temporal changes in imaging availability, they cannot fully account for differences in surveillance intensity or diagnostic thresholds. Residual detection bias should therefore be considered when interpreting these findings. Finally, detailed systemic treatment data were not uniformly available across the full study period, including the use of PARP inhibitors and other agents with potential intracranial activity. Given the established efficacy of PARP inhibitors in gBRCA-associated metastatic breast cancer (e.g., OlympiAD, EMBRACA), unmeasured differences in treatment exposure may have influenced survival outcomes across groups. This is particularly relevant for the time-period analyses, as the uptake of targeted therapies has increased over time. As such, residual confounding by treatment cannot be excluded. Future studies with detailed treatment data will be important to determine whether differences in systemic therapy contribute to the observed survival patterns.

## 5. Conclusions

In this multicenter retrospective cohort, g*BRCA2* PV carriers demonstrated longer unadjusted survival after CNS metastasis and a longer interval to CNS involvement compared with g*BRCA1* PV carriers and non-carriers. However, these associations were attenuated after adjustment for molecular subtype and clinical factors and should be interpreted as exploratory rather than evidence of an independent prognostic effect. Larger prospective cohorts with standardized CNS endpoints and detailed treatment data are required to validate these findings.

## Figures and Tables

**Table 1:** Overall characteristics of 115 patients categorized by g*BRCA* status.

	g*BRCA1* PV N (%) 32 (28)	g*BRCA2* PV N (%) 18 (16)	Non-Carriers N (%) 65 (57)	*p*-Value ^1^
Age at CNSmet diagnosis, median [IQR]	41.1 [39.1, 50.1]	48.9 [45.0, 56.8]	51.6 [41.1, 60.8]	**0.003**
Menopausal status at diagnosis				0.119
Premenopausal	3 (9.4)	2 (11.1)	9 (13.8)	
Postmenopausal	19 (59.4)	9 (50.0)	20 (30.8)	
Unknown	10 (31.2)	7 (38.9)	36 (55.4)	
BC histology				0.258
DCIS	0 (0.0)	0 (0.0)	4 (6.2)	
IDC	29 (90.6)	16 (88.9)	44 (67.7)	
ILC	0 (0.0)	1 (5.6)	5 (7.7)	
Inflammatory	0 (0.0)	0 (0.0)	1 (1.5)	
Unknown	3 (9.4)	1 (5.6)	11 (16.9)	
BC molecular subtype				0.053
Luminal A	2 (6.2)	5 (27.8)	11 (16.9)	
Luminal B	2 (6.2)	3 (16.6)	6 (10.8)	
HER2+	0 (0.0)	0 (0.0)	5 (7.7)	
TN	21 (65.6)	3 (16.7)	33 (50.8)	
Unknown	7 (21.9)	7 (38.9)	9 (13.8)	
BC therapy				
Chemotherapy	28 (87.5)	15 (83.3)	51 (78.5)	0.546
Radiotherapy	24 (75.0)	13 (72.2)	44 (67.7)	0.747
Surgery	32 (100.0)	17 (94.4)	52 (80.0)	**0.012**
Number of CNSmet				0.845
1	6 (18.8)	4 (22.2)	20 (30.8)	
≥2	22 (68.8)	11 (61.1)	37 (56.9)	
Unknown	4 (12.5)	3 (16.7)	8 (12.3)	
Leptomeningeal disease	15 (46.9)	6 (33.3)	26 (40.0)	0.631
CNSmet as first metastatic site	15 (46.9)	9 (50.0)	27 (41.5)	0.770
Singular CNSmet ^2^	7 (21.9)	5 (27.8)	8 (12.3)	0.227
Other sites of metastasis				
Lung	13 (40.6)	5 (27.8)	36 (55.4)	0.081
Liver	11 (34.4)	4 (22.2)	26 (40.0)	0.373
Bone	13 (40.6)	11 (61.1)	32 (49.2)	0.377
Lymph Nodes	10 (31.2)	4 (22.2)	31 (47.7)	0.082
Skin	3 (9.4)	1 (5.6)	10 (15.4)	0.450
Other ^3^	1 (3.1)	1 (5.6)	13 (20.0)	0.040
Number of distant metastases sites (excluding CNSmet), median [IQR]	1.0 [1.0, 2.3]	1.5 [0.3, 2.0]	2.00 [1.00, 3.00]	**0.014**
CNSmet therapy				0.527
WBRT	12 (37.5)	9 (50.0)	18 (27.7)	
SRS	4 (12.5)	2 (11.1)	10 (15.4)	
SRS + WBRT	5 (15.6)	1 (5.6)	7 (10.8)	
Surgery	0 (0.0)	2 (11.1)	3 (4.6)	
Surgery + WBRT	2 (6.2)	1 (5.6)	10 (15.4)	
Surgery + SRS + WBRT	4 (12.5)	1 (5.6)	4 (6.2)	
None	5 (15.6)	2 (11.1)	13 (20.0)	

Abbreviations: BC, breast cancer; CNSmet, CNS metastasis; DCIS, ductal carcinoma in situ; IDC, invasive ductal carcinoma; ILC, invasive lobular carcinoma; TN, triple negative; SRS, stereotactic radiosurgery; WBRT, whole-brain radiation therapy; SD, standard deviation; IQR, interquartile range. *p*-values in bold: significant (*p* < 0.05). ^1^ Kruskal–Wallis test for continuous variables and Chi-squared or Fisher’s exact test for categorical variables; unknown category is excluded from comparison. ^2^ Patients who presented with CNS metastases only (no other metastatic site). ^3^ Other sites of metastases recorded were gastrointestinal (7), thyroid (1), bladder (1), adrenal gland (2), orbita (1), connective soft tissue (1), mediastinum (2).

**Table 2:** Subtype-stratified Cox proportional hazards regression *.

	HR	95% CI (Lower–Upper)	*p*-Value
Age at CNSmet Diagnosis (per year)	1.00	0.97–1.02	0.776
Number of metastases			
1	1.00	-	-
≥2	1.86	0.94–3.71	0.076
Leptomeningeal disease	1.25	0.801.97	0.326
No	1.00		
Yes	1.50	0.89–2.54	0.130
Extracranial metastases			
No	1.00		
Yes	1.32	0.54–3.24	0.540
g*BRCA* status			
Non-carrier	1.00	-	-
g*BRCA1*	0.90	0.49–1.64	0.730
g*BRCA2*	0.48	0.18–1.25	0.131
CNS-directed therapy			
WBRT only	1.00	-	-
SRS only	0.55	0.23–1.30	0.175
SRS + WBRT	0.57	0.26–1.28	0.173
Any surgery (±WBRT/SRS)	0.58	0.27–1.26	0.164
No local therapy	2.28	0.84–6.18	0.105

Abbreviations: HR, hazard ratio; CI, confidence interval; CNS, central nervous system; SRS, stereotactic radiosurgery; WBRT, whole-brain radiation therapy. * No evidence of proportional hazards violations was observed based on Schoenfeld residuals test. Complete-case N = 79 (gBRCA1 PV = 23, gBRCA2 PV: 10, non-carrier: 46), with 69 deaths and 10 censored.

## Data Availability

Data is available upon reasonable request from the corresponding author.

## Supplementary Materials

The following supporting information can be downloaded at: https://www.mdpi.com/article/10.3390/cancers18081240/s1, Figure S1: Overall survival post-CNS metastasis (MUV); Figure S2: Overall survival post-CNS metastasis (kConFab); Table S1. Overall characteristics by cohort; Table S2. Cohort-specific univariable Cox models—OS from CNS metastasis diagnosis. Table S3. Cox model, subtype as a covariate; Table S4. Subtype-stratifed Cox model + treatment time-period; Table S5. Cox model, subtype as covariate + treatment time-period.

## Abbreviations

BC	breast cancer
CNS	central nervous system
CNSmet	central nervous system metastasis
CT	computer tomography
DCIS	ductal carcinoma in situ
ER	estrogen receptor HER2/ERBB human epithelial growth factor receptor 2
HRD	homologous recombination deficiency
IDC	invasive ductal carcinoma
ILC	invasive lobular carcinoma
kConFab	Kathleen Cuningham Foundation Consortium for research into familial breast cancer
LCIS	lobular carcinoma in situ
MRI	magnetic resonance imaging
MUV	Medical University of Vienna/Vienna General Hospital
PV	pathogenic variant
TNBC	triple-negative breast cancer
VUS	variant of uncertain significance
WBRT	whole-brain radiotherapy
