# Supplementary material for: Gene-Specific Outcomes After Central Nervous System Metastases in Germline BRCA1- and BRCA2-Associated Breast Cancer
Source: Cancers (Basel). 2026 Apr 14;18(8):1240. doi: 10.3390/cancers18081240 (PMC13114316; doi:10.3390/cancers18081240)
Supplement: Supplementary file 1 [file cancers-18-01240-s001.zip › cancers-4226171-supplementary.pdf]

Supplementary Table S1. Overall characteristics by cohort

	Overall N=115	MUV N=58	kConFab N=57	p-value[1]
**Age at CNSmet diagnosis, median [IQR]**	48.4 [40.3, 56.3]	47.9 [39.1, 55.7]	49.4 [41.1, 57.0]	0.258
	**N (%)**	**N (%)**	**N (%)**	
**BRCA status**				**0.007**
g*BRCA1*	32 (27.8)	12 (20.7)	20 (35.1)	
g*BRCA2*	18 (15.7)	5 (8.6)	13 (22.8)	
Non-carrier	65 (56.5)	41 (70.7)	24 (42.1)	
**Sex assigned at birth**				0.595
Female	105 (91.2)	57 (98.3)	48 (84.2)	
Male	3 (2.6)	1 (1.7)	2 (3.5)	
Unknown	7 (6.1)	0 (0.0)	7 (12.3)	
**Menopausal status**				**0.044**
Premenopausal	48 (41.7)	10 (17.2)	38 (66.7)	
Postmenopausal	14 (12.2)	7 (12.1)	7 (12.3)	
Unknown	53 (46.1)	41 (70.7)	12 (21.1)	
**BC Histology**				0.115
DCIS	4 (3.5)	4 (6.9)	0 (0.0)	
IDC	89 (77.4)	46 (79.3)	43 (75.4)	
ILC	6 (5.2)	2 (3.4)	4 (7.0)	
Inflammatory	1 (0.9)	1 (1.7)	0 (0.0)	
Unknown	15 (13.0)	5 (8.16)	10 (17.5)	
**BC Laterality**				0.116
Left	60 (52.2)	33 (56.9)	27 (47.4)	
Right	42 (36.5)	19 (32.8)	23 (40.4)	
Bilateral	4 (3.5)	4 (6.9)	0 (0.0)	
Unknown	9 (7.8)	2 (3.4)	7 (12.3)	
**BC molecular subtype**				**<0.001**
Luminal A	18 (15.7)	5 (8.6)	13 (22.8)	
Luminal B	12 (10.4)	7 (12.1)	5 (8.8)	
HER2+	5 (4.3)	2 (3.4)	3 (5.3)	
TN	57 (49.6)	44 (75.9)	13 (22.8)	
Unknown	23 (20.0)	0 (0.0)	24 (41.4)	
**BC Therapy**				
Chemotherapy	94 (81.7)	53 (91.4)	41 (71.9)	**0.014**
Radiotherapy	81 (70.4)	43 (74.1)	38 (66.7)	0.501
Surgery	101 (87.8)	52 (89.7)	49 (86.0)	0.749
**Number of CNSmet**				0.192
1	30 (26.1)	16 (27.6)	14 (24.6)	
≥2	70 (60.9)	39 (67.2)	31 (54.4)	
Unknown	15 (13.0)	3 (5.2)	12 (21.1)	
**Leptomeningeal Disease**	47 (40.9)	24 (41.4)	23 (40.4)	>0.999
**Diagnostic tool for CNSmet**				**<0.001**
MRI	75 (65.2)	50 (86.2)	25 (43.9)	
CT	24 (20.9)	4 (6.9)	20 (35.1)	
Lumbar puncture	3 (2.6)	1 (1.7)	2 (3.5)	
Unknown	13 (11.3)	3 (5.2)	10 (17.5)	

**CNSmet as first metastatic site**	51 (44.3)	27 (46.6)	24 (42.1)	0.770
**Singular CNSmet[2]**	20 (17.4)	9 (15.5)	11 (19.3)	0.773
**Number of distant metastases sites (excluding CNSmet), median [IQR]**	2.0 [1.0, 3.0]	2.0 [1.0, 3.0]	2.0 [1.0, 3.0]	0.817
**Other Sites of Metastasis**				
Lung	54 (47.0)	29 (50.0)	25 (43.9)	0.636
Liver	41 (35.7)	17 (29.8)	24 (43.6)	0.216
Bone	56 (48.7)	22 (3.6)	34 (65.4)	**0.032**
Lymph Nodes	45 (39.1)	32 (55.2)	13 (24.1)	**0.001**
Skin	14 (12.2)	9 (15.8)	5 (9.8)	0.412
Other[3]	15 (13.2)	2 (3.5)	13 (24.1)	**0.005**
**CNSmet therapy**				**<0.001**
SRS	13 (13.9)	12 (20.7)	4 (7.0)	
WBRT	39 (33.9)	13 (22.8)	26 (45.6)	
Surgery	5 (4.3)	1 (1.8)	3 (6.0)	
SRS + WBRT	13 (11.3)	11 (19.0)	2 (3.5)	
Surgery + WBRT	13 (11.3)	11 (19.3)	2 (4.0)	
Surgery + SRS + WBRT	9 (7.8)	5 (8.6)	4 (7.0)	
None	20 (17.4)	5 (8.6)	15 (26.3)	

Abbreviations:
BC: Breast Cancer; **CNSmet**: CNS Metastasis; **DCIS**: Ductal Carcinoma in Situ; **IDC**: Invasive Ductal Carcinoma; **ILC**: Invasive Lobular Carcinoma; **TN** triple negative; **MRI**: Magnetic Resonance Imaging; **CT**: Computer Tomography; **SRS** Stereotactic Radiosurgery; **WBRT**: Whole brain radiation therapy; SD standard deviation; IQR interquartile range
p-values in **bold**: significant (p<0.05)
[1] Kruskal-Wallis or Chi-square or Fisher's exact test, where appropriate; unknown category excluded from comparison
[2]Patients who presented with CNS metastases only (no other metastatic site)
[3]Other sites of metastases recorded were: Gastrointestinal (7), Thyroid (1), Bladder (1), adrenal gland (2), Orbita (1), Connective soft tissue (1), Mediastinum (2)

Supplementary Table S2. Cohort-specific univariable Cox models – OS from CNS metastasis diagnosis*

Cohort	Comparison	N	Deaths	Censored	HR	95% CI	p
MUV	g*BRCA1* PV	58	49	9	0.96	0.49–1.87	0.893
	g*BRCA2* PV	58	49	9	0.26	0.06–1.09	0.065
kConFab	g*BRCA1* PV	57	55	2	1.28	0.70–2.34	0.424
	g*BRCA2* PV	57	55	2	0.62	0.29–1.33	0.220
* Reference group = non-carriers. Models were fitted separately within each cohort with germline status as the only predictor (gBRCA1 PV vs noncarrier and gBRCA2 PV vs noncarrier). Hazard ratios are shown with 95% confidence intervals							

Supplementary Table S3. Cox model, subtype as a covariate

	HR	95% CI (lower-upper)	p-value
**Age at CNSmet Diagnosis** (per year)	1.00	0.97-1.02	0.704
**Molecular subtype**			

Luminal	1.00		
TNBC	1.83	1.00-3.36	**0.050**
**Number of metastases**			
1	1.00	-	-
≥2	1.91	0.98-3.70	0.056
**Leptomeningeal disease**	1.25	0.80-1.97	0.326
No	1.00		
Yes	1.50	0.89-2.54	0.125
**Extracranial metastases**			
No	1.00		
Yes	1.40	0.57-3.42	0.464
**gBRCA status**			
WT	1.00	-	-
gBRCA1	0.90	0.49-1.64	0.722
gBRCA2	0.46	0.18-1.17	0.104
**CNS-directed therapy**			
WBRT only	1.00	-	-
SRS only	0.55	0.23-1.30	0.174
SRS + WBRT	0.61	0.27-1.35	0.222
Any surgery (±WBRT/SRS)	0.62	0.29-1.35	0.227
No local therapy	2.25	0.84-6.08	0.108

Abbreviations:

HR hazard ratio; **CI** confidence interval; **CNS** central nervous system; **SRS** stereotactic radiosurgery; **WBRT** whole brain radiation therapy

Supplementary Table S4. Subtype-stratifed Cox model + treatment time-period

	HR	95% CI (lower-upper)	p-value
**Age at CNSmet Diagnosis** (per year)	1.00	0.97-1.02	0.814
**Number of metastases**			
1	1.00	-	-
≥2	1.69	0.83-3.46	0.149
**Leptomeningeal disease**	1.25	0.801.97	0.326
No	1.00		
Yes	1.39	0.80-2.41	0.244
**Extracranial metastases**			
No	1.00		
Yes	1.50	0.59-3.83	0.397
**gBRCA status**			
WT	1.00	-	-
gBRCA1	0.97	0.52-1.81	0.933
gBRCA2	0.57	0.20-1.57	0.277
**CNS-directed therapy**			
WBRT only	1.00	-	-
SRS only	0.52	0.21-1.22	0.132
SRS + WBRT	0.57	0.25-1.27	0.169
Any surgery (±WBRT/SRS)	0.56	0.26-1.22	0.142
No local therapy	2.21	0.82-5.98	0.117
**Treatment time-period**			
Pre-2010	1.00		
Post-2010	1.96	0.46-8.45	0.365

Abbreviations:
HR hazard ratio; **CI** confidence interval; **CNS** central nervous system; **SRS** stereotactic radiosurgery; **WBRT** whole brain radiation therapy

Supplementary Table S5. Cox model, subtype as covariate + treatment time-period

	HR	95% CI (lower-upper)	p-value
**Age at CNSmet Diagnosis** (per year)	1.00	0.97-1.02	0.764
**Molecular subtype**			
Luminal	1.00		
TNBC	2.06	1.06-3.99	**0.033**
**Number of metastases**			
1	1.00	-	-
≥2	1.71	0.85-3.46	0.134
**Leptomeningeal disease**	1.25	0.801.97	0.326
No	1.00		
Yes	1.40	0.81-2.42	0.227
**Extracranial metastases**			
No	1.00		
Yes	1.57	0.62-3.98	0.346
**gBRCA status**			
WT	1.00	-	-

gBRCA1	0.96	0.52-1.78	0.893
gBRCA2	0.55	0.20-1.53	0.252
**CNS-directed therapy**			
WBRT only	1.00	-	-
SRS only	0.52	0.22-1.23	0.138
SRS + WBRT	0.60	0.27-1.35	0.215
Any surgery (±WBRT/SRS)	0.60	0.28-1.31	0.201
No local therapy	2.22	0.83-5.98	0.114
**Treatment time-period**			
Pre-2010	1.00		
Post-2010	1.78	0.46-6.85	0.400

Abbreviations:
HR hazard ratio; **CI** confidence interval; **CNS** central nervous system; **SRS** stereotactic radiosurgery; **WBRT** whole brain radiation therapy

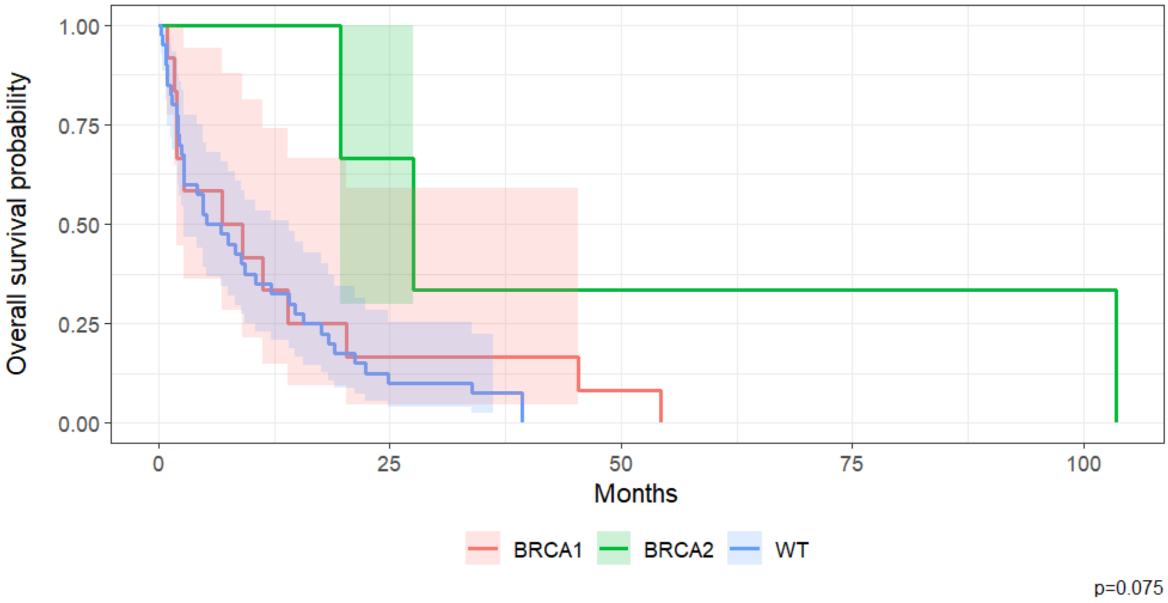

At Risk					
BRCA1	12	2	1	0	0
BRCA2	5	2	1	1	1
WT	41	4	0	0	0
**Events**					
BRCA1	0	10	11	12	12
BRCA2	0	1	2	2	2
WT	0	36	38	38	38

Supplementary Figure S1: Overall survival post-CNS metastasis (MUV)

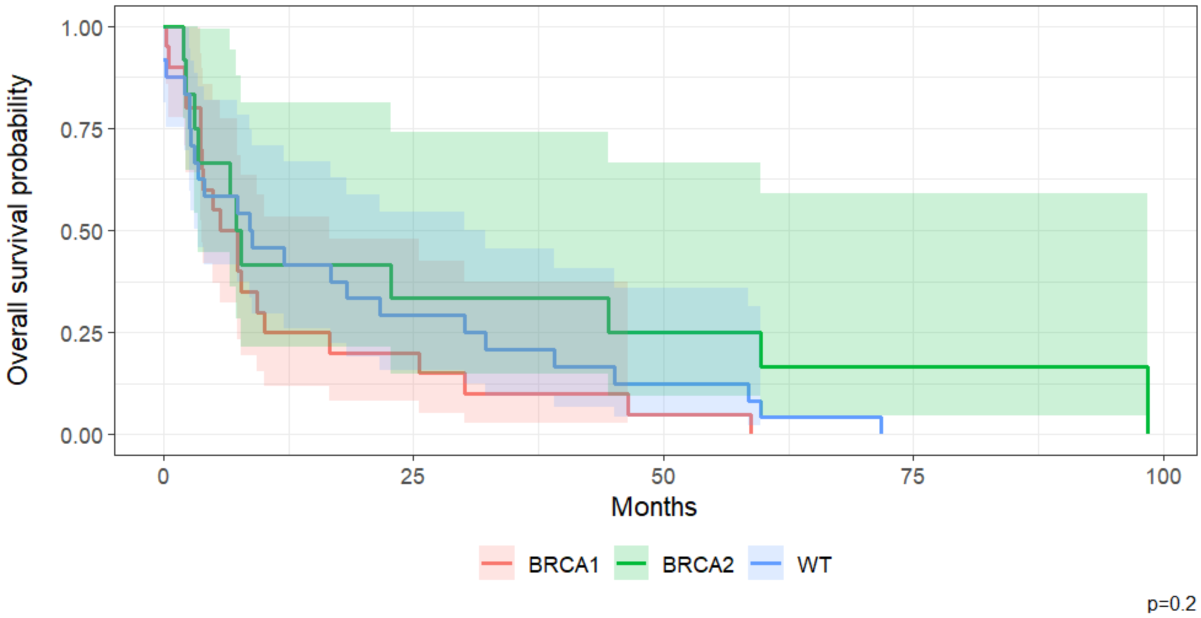

	0	25	50	75	100
**At Risk**					
BRCA1	20	4	1	0	0
BRCA2	13	4	3	2	0
WT	24	7	3	0	0
**Events**					
BRCA1	0	16	19	20	20
BRCA2	0	8	9	10	11
WT	2	17	21	24	24

Supplementary Figure S2: Overall survival post-CNS metastasis (kConFab).
